# Deep soil C and N pools in long-term fenced and overgrazed temperate grasslands in northwest China

**DOI:** 10.1038/s41598-019-52631-6

**Published:** 2019-11-06

**Authors:** Jian-Ping Li, Hong-Bin Ma, Ying-Zhong Xie, Kai-Bo Wang, Kai-Yang Qiu

**Affiliations:** 1 School of Agriculture, Ningxia University, Yinchuan, China; 2 State Key Laboratory of Loess and Quaternary Geology, Institute of Earth Environment, Chinese Academy of Sciences, Xi’an, China

**Keywords:** Conservation biology, Grassland ecology

## Abstract

Fencing for grazing exclusion has been widely found to have an impact on grassland soil organic carbon (SOC) and total nitrogen (TN), but little is known about the impact of fenced grassland on the changes in deep soil carbon (C) and nitrogen (N) stocks in temperate grasslands. We studied the influence of 30 years fencing on vegetation and deep soil characteristics (0–500 cm) in the semi-arid grasslands of northern China. The results showed that fencing significantly increased the aboveground biomass (AGB), litter biomass (LB), total biomass, vegetation coverage and height, and soil water content and the SOC and TN in the deep soil. The belowground biomass (BGB) did not significantly differ between the fenced and grazed grassland. However, fencing significantly decreased the root/shoot ratio, forbs biomass, pH, and soil bulk density. Meanwhile, fencing has significantly increased the C and N stocks in the AGB and LB but not in the BGB. After 30 years of fencing, the C and N stocks significantly increased in the 0–500 cm soil layer. The accumulation of SOC mainly occurred in the deep layers (30–180 cm), and the accumulation of TN occurred in the soil layers of 0 to 60 cm and 160 to 500 cm. Our results indicate that fencing is an effective way to improve deep soil C and N stocks in temperate grassland of northwest China. There were large C and N stocks in the soil layers of 100 to 500 cm in the fenced grasslands, and their dynamics should not be ignored.

## Introduction

Soil carbon (C) stocks have an important feedback effect on global climate change^1^; small changes in soil C content over large areas can substantially intensify or mitigate current increases in atmospheric CO_2_^2,3^. Grasslands covered about 25% of the Earth’s land area and 10% of global C stocks and are thus vital to global C cycling^4^. Grasslands contain a large amount of C and nitrogen (N)^5^, which are important for soil health and biomass production because soil organic matter improves the soil water holding capacity, nutrient cycling and soil structure^6^.

Overgrazing has a negative influence on plant biomass, plant diversity, and soil C accumulation in grasslands^7–9^, especially in arid and semi-arid grasslands^8^. Many types of grassland have become degraded as a result of overgrazing, which may lead to grassland desertification^10,11^. Moreover, overgrazing might decrease the C and N pools of grassland ecosystems^8,12^. Many studies have found that more than 10 years of grazing exclusion facilitated vegetation recovery and increased plant productivity and thus enhanced soil C stocks and N stocks in degraded grasslands^7,13–15^. Accordingly, grazing exclusion by fencing is a common practice for restoring overgrazed grasslands^16^.

In China, approximately 40% of the total land area is covered by grasslands, the grassland areas account for approximately 6–8% of the total global grassland area, and their C stocks account for 9–16% of the world’s total grassland C stocks^17^. However, over 90% of grasslands had been widely degraded by the end of the twentieth century^18^ due to long-term overgrazing^19^. Over the past 30 years, fencing has been widely adopted in China to restore degraded rangelands, and a series of improved grassland management strategies have been implemented. A nationwide conservation project, Returning Grazing Lands to Grasslands (RGLG), was implemented in 2003 to restore vegetation and soil, and fencing has been the most common practice and most effective method of restoring degraded rangelands in northwest China. Previous studies have detected an increase in soil organic carbon (SOC) stocks (top 30 cm of soil) in fenced grasslands on the Tibetan Plateau^20^ and accumulated soil C and N stocks (0–100 cm) in natural restoration grasslands on the Loess Plateau^21^. However, comparably less SOC and total nitrogen (TN) information is available for deep soil (100–500 cm), and SOC and TN in the deep soil is often ignored.

In this study, we used the “space-for-time” substitution technique as the main method to study the evolution of ecosystem properties over time in the Yunwu Mountain reserve. The reserve, fencing for grazing exclusion since 1982, is temperate grassland of the Loess Plateau. The reserve is a better location for understanding the influence of fencing on grassland vegetation, deep soil characteristics, and the C and N pools. Before the fencing was placed for grazing exclusion, the grasslands were used as grazing land, the site original condition (plant diversity and soil properties) were almost same in both the grazed and fenced grasslands^22^, Therefore, the existing grazed grassland is suitable for control to compare the long-term fencing grassland (30 years) on grassland vegetation and deep soil characteristics.

The overall objective of this work was to figure out the influences of grazing exclusion on the (i) vegetation characteristics, (ii) soil physiochemical properties in the deep soil (0–500 cm), and (iii) the C and N stocks in the deep soil layers. The research reveals plant productivity, grassland ecological recovery and deep soil C and N change in the semi-arid grasslands of northern China.

## Results

### Plant properties

The fenced grassland (FG) had greater aboveground biomass (AGB) (P < 0.001), litter biomass (LB) (P < 0.05), total biomass (TB) (P < 0.001), soil coverage (P < 0.001) and plant height (P < 0.001) than the grazed grassland (GG). The belowground biomass (BGB) (P = 0.0535) in the underlying soils was not significantly different between the FG and GG (Table 1). However, the FG had lower values for the root/shoot ratio (R/S) (P < 0.001) and forbs (P < 0.001). Thus, after 30 years of fencing (fencing started in 1984), the TB of the grassland increased significantly from 808.3 g m^−2^ to 1371.2 g m^−2^, the fraction of forbs decreased dramatically from 47.6% to 16.8%, and the R/S ratio doubled.

**Table 1:** Plant properties of the fenced (FG) and grazed (GG) grassland communities (n = 3).

Grasslands	AGB (g m^−2^)	BGB (g m^−2^)	LB (g m^−2^)	TB (g m^−2^)	R/S	Coverage (%)	Height (cm)	Grass (%)	Forb (%)
FG	659.8 ± 26.9	637.8 ± 36.0	73.6 ± 5.8	1371.2 ± 55.9	1.0 ± 0.01	97.4 ± 0.7	56.0 ± 1.1	83.2 ± 3.7	16.8 ± 3.7
GG	233.9 ± 14.4	523.0 ± 42.9	51.4 ± 6.9	808.3 ± 49.5	2.3 ± 0.2	45.9 ± 3.3	21.0 ± 1.9	52.4 ± 9.0	47.6 ± 9.0
Sig.	***	NS	*	***	***	***	***	***	***

Note: AGB, aboveground biomass; BGB, belowground biomass; LB, litter biomass; TB, total biomass; R/S, root/shoot ratio. The values (mean ± SD) are the means of three samples; significant differences between fenced and grazed grasslands are indicated by the following symbols: ***P < 0.001, **P < 0.01, *P < 0.05. NS denotes no significant difference.

### Soil physical and chemical properties

**Figure 1:**
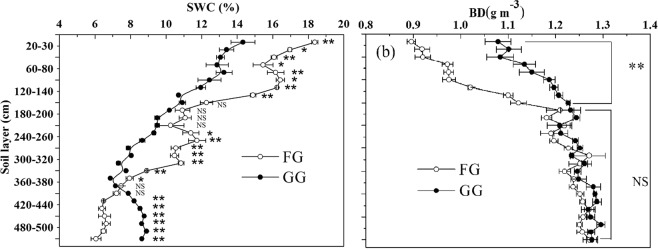
(**a**) Soil water content (SWC) and (**b**) soil bulk density (BD) in fenced grassland (FG) and grazed grassland (GG). Note: the values are mean ± SD; difference significant was represented by asterisk (P < 0.05, denoted by *P < 0.01, denoted by **P > 0.05, denoted by NS).

**Figure 2:**
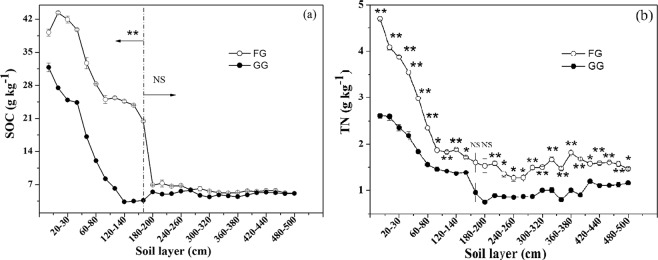
(**a**) Soil organic carbon (SOC) and (**b**) soil total nitrogen (TN) in fenced grassland (FG) and grazed grassland (GG). Note: the values are mean ± SD; difference significant was represented by asterisk(P < 0.05, denoted by *P < 0.01, denoted by **P > 0.05, denoted by NS).

Fencing improved the soil water content (SWC) in the soil layer of 0 to 360 cm of the FG and led to significant differences between the FG and GG in the 0–120 cm and 200–360 cm soil layers (P < 0.05) (Fig. 1a). In the 360–500 cm soil layer, the SWC in the FG was lower than that in the GG(P < 0.01). Meanwhile, fencing significantly decreased the soil bulk density (BD) of the soil layer of 0 to 140 cm (P < 0.01) but not the 140–500 cm soil layer (P > 0.05), and the BD of FG had not significant difference from GG for the soil layers (Fig. 1b). There was no difference in the SOC of the 180–500 cm soil layer between the FG and GG areas (P > 0.05), but the SOC was greater in the soil layers from 0 to 180 cm in the FG (P < 0.001) (Fig. 2a). In addition, Fencing for grazing exclusion significantly increased the soil TN stocks in all soil layers from 0 to 500 cm (P < 0.05), except the soil layers of 160 to 200 cm (Fig. 2b).

### Plant C and N pools

**Figure 3:**
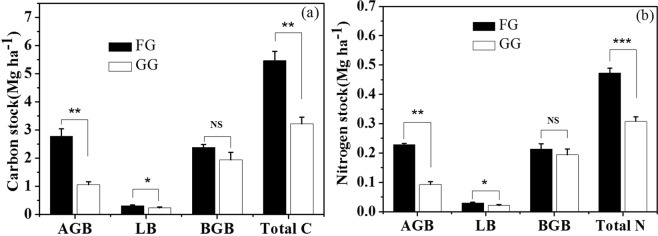
(**a**) C and (**b**) N stock of AGB, LB, BGB and total vegetation in fenced grassland (FG) and grazed grassland (GG). AGB, LB and BGB represent aboveground biomass, litter biomass and belowground biomass, respectively. Note: the values are mean ± SD; difference significant was represented by asterisk(P < 0.05, denoted by *P < 0.01, denoted by **P < 0.001, denoted by ***P > 0.05, denoted by NS).

**Figure 4:**
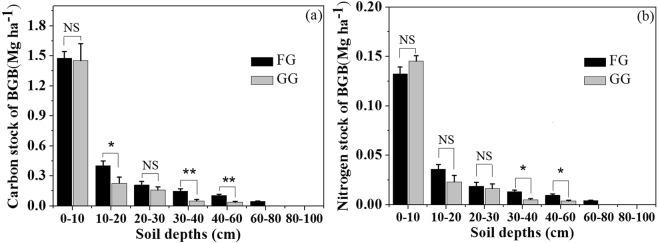
(**a**) C stock and (**b**) N stock of belowground biomass (BGB) in different soil layers of fenced grassland (FG) and grazed grassland (GG). Note: the values are mean ± SD; difference significant was represented by asterisk (P < 0.05, denoted by *P > 0.05, denoted by NS).

Fencing for grazing exclusion improved the C stock in the AGB and LB significantly (Fig. 3a). The C stocks in the AGB and LB were 2.7 times (P < 0.01) and 1.4 times (P < 0.05) greater in the FG than in the GG, respectively; the C stock of the plant in the FG was 70.1% greater than that in the GG (P < 0.01); and the C stock of the BGB in the FG was not significantly different from that in the GG (P > 0.05). While the N stocks of the AGB and LB in the FG were 146.9% and 41.2% greater (P < 0.05) than those in the GG, respectively (Fig. 3b), the total plant N stock in the FG was 50.6% greater than that in the GG (P < 0.001), but the N stock of the BGB in the FG was not significantly different from that in the GG (P > 0.05). In the soil layer from 30 to 60 cm, the C and N stocks of the BGB in the FG were significantly greater than those in the GG (Fig. 4). However, in the soil layers of 60 to 100 cm, the C and N stock of BGB in the FG was not significantly different from that in the GG (P > 0.05) (Fig. 4). In the soil layer from 0 to 10 cm and 20 to 30 cm, the C stock of BGB in the FG was not significantly different from that in GG, but in the soil layer from 10 to 20 cm, the C stocks of the BGB in the FG were significantly greater than those in the GG (Fig. 4a). The N stock of the BGB in the soil layers from 0 to 30 cm did not differ between the FG and GG (P > 0.05) (Fig. 4b).

### Soil C and N pool

**Figure 5:**
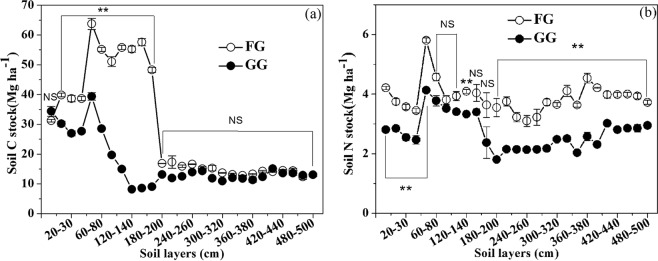
(**a**) Soil C and (**b**) N stock in different soil layers of fenced grassland (FG) and grazed grassland (GG). Note: the values are mean ± SD; difference significant was represented by asterisk (P < 0.05, denoted by *P < 0.01, denoted by **P < 0.001, denoted by ***P > 0.05, denoted by NS).

Long-term fencing significantly increased soil C in the soil layers of 10 to 180 cm (P < 0.01), while non-significant increases were observed in the 0 to 10 cm and 180 to 500 cm soil layers (P > 0.05) (Fig. 5a). Meanwhile, fencing significantly increased the soil N stock both in the 0 to 60 cm (P < 0.01; Fig. 5b) and 200 to 500 cm soil layers (P < 0.01; Fig. 5b), but non-significant increases were observed for the 80 to 120 cm and 140 to 180 cm soil layers (P > 0.05) (Fig. 5b).

**Figure 6:**
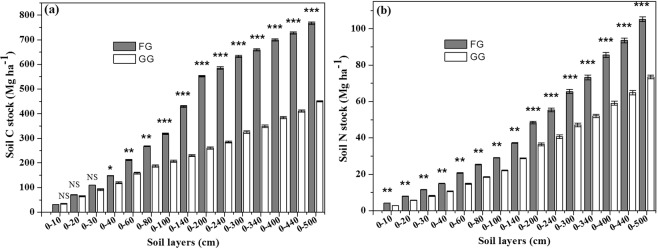
(**a**) Cumulative soil C storage and (**b**) cumulative soil N storage in fenced grassland (FG) and grazed grassland (GG). Note: the values are mean ± SD; difference significant was represented by asterisk (P < 0.05, denoted by *P < 0.01, denoted by **P < 0.001, denoted by ***P > 0.05, denoted by NS).

The accumulation of soil carbon storage in the 0–30 cm layer of soil did not significantly differ between the FG and GG (P > 0.05); the cumulative soil C stock in the 0–40 cm (P < 0.05), 0–60 cm (P < 0.01) and 0–80 cm (P < 0.01) soil layers significantly increased in the FG compared to GG, respectively; and the accumulation of soil carbon in the soil profile of 0–100, 0–140, 0–200, 0–240, 0–300, 0–340, 0–400, 0–440, and 0–500 cm were significantly greater in the FG than in the GG (Fig. 6a). The N storage for 0–500 cm in the GG was greater than in the FG (for the 0–180 and 180–500 cm soil layers, both P < 0.01) (Fig. 6b).

**Figure 7:**
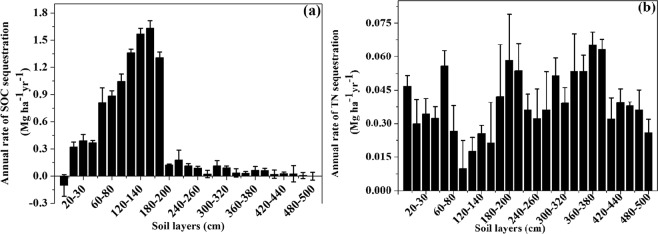
Annual rates of (**a**) SOC stock and (**b**) TN sequestration.

After 30 years of fencing, the grassland accumulated SOC in different soil layers; however, in the 0–10 cm soil layer, the soil C stock sequestration showed a negative value, indicating that the FG had lost C from soil over the past 30 years. The annual rate of soil C stock sequestration increased greatly from the 40 to 180 cm soil layer(Fig. 7a); however, for the 180–500 cm soil layer, the soil stock sequestration was low. The annual rates of TN sequestration were high in the 0–60 cm soil layers and 160–500 cm soil layers (Fig. 7b).

### Grassland ecosystem C and N pools

**Figure 8:**
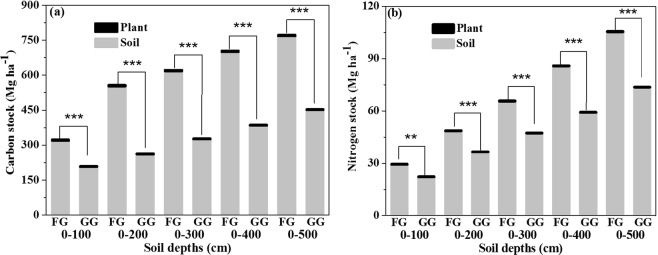
(**a**) Ecosystem carbon (C) and (**b**) nitrogen (N) pools in different soil layers. Note: values are the mean ± SD; significant differences in the C or N pools between the FG and GG treatments are indicated by asterisk (P < 0.01, denoted by **P < 0.001, denoted by***).

The ecosystem C storages in the fenced grassland were significantly greater than those in the grazed grassland (P < 0.001, Fig. 8a) in all soil layers over the 30 years of the experiment, and the plant C stocks represented a small fraction of the ecosystem C stocks. In addition to the ecosystem C pool, the ecosystem N pool in the FG significantly increased in every soil layer (0–100 cm, P < 0.01; 0–200, 0–300, 0–400 and 0–500 cm, P < 0.001) in comparison to that in the GG (Fig. 8b). Using data from Fig. 8, we estimated that the ecosystem C stocks in the 0–100 cm soil layer of the FG accounted for 41.8% and 58.1% of the ecosystem C stocks in the 0–500 cm and 0–200 cm soil layers, respectively. Meanwhile, the ecosystem N stocks in the 0–100 cm soil layers of the FG accounted for 28.0% and 60.6% of the ecosystem C stocks in the 0–500 cm and 0–200 cm soil layers, respectively.

## Discussion

In this study, we found that fencing had positive effects on vegetation cover, biomass and height, as also reported in other studies^12,16,23,24^. Fencing can enhance plant cover, biomass and height because it protects the soil seed bank and increases species composition recovery^16,25^. Moreover, this study showed that grazing exclusion had weak effects on the BGB in comparison to that in the GG. Conversely, previous studies have supported the hypothesis that grazing exclusion has negative effects on BGB in the 1 m soil layer^12,26^ or at least has no detrimental effects^27^ but reduces the percentage of forbs; the forb fraction also decreased in our study. The main factor that affected the plant properties in the grazed grassland: standing plant biomass was continuously removed by herbivory, after which the litter was easily lost^28–30^; this scenario would then decrease the AGB and LB. Oppositely, fencing may increase in soil coverage, plant diversity, vegetation biomass, SWC and SOC, which would increase the AGB and LB^31,32^.

**Figure 9:**
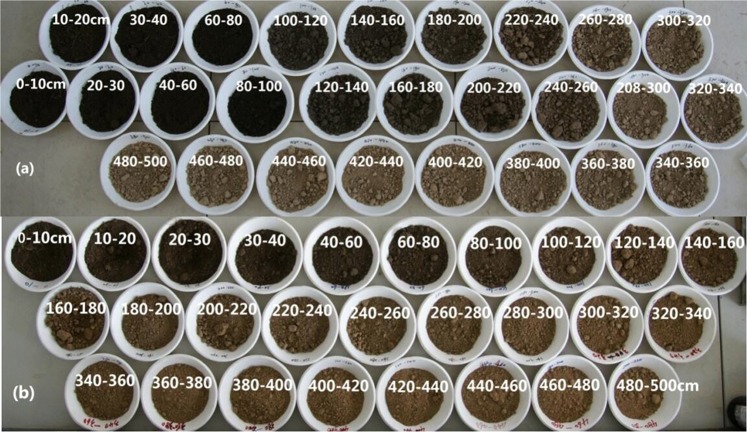
Soil samples in (**a**) FG and (**b**) GG from 0 to 500 cm.

This study showed that fencing had significant effects on SWC, BD, SOC and soil TN. Fencing increased the SWC in the 0–140 cm soil layer because the high coverage of vegetation and more plant litter may have improved the soil moisture retention and protected the soil water from evaporation^23,33^. Meanwhile, the soil BD in the 0–140 soil layer decreased in the FG, likely because trampling may have increased the BD in the GG but not in the FG, and an increase in plant roots and soil microorganisms may have decreased the BD in the FG^33–35^. In our study, in the soil layer from 0 to 180 cm, the SOC in the FG was greater than that in the GG, and fencing significantly increased the soil TN storage in the soil layers of 0 to 500 cm (P < 0.05). The soil in the 0 to 180 cm layer was black in the FG (Fig. 9a); this relatively deeper colour likely corresponded to a greater SOC fraction in the FG soil than in the GG soil (Fig. 9b). Previous studies showed that grazing exclusion result in significant increasing of SOC and TN as a result of perennial organic matter inputs from plant decomposition, and the lack of disturbance and formation of SOC in micro aggregates lead to the creation of fine soil particles, which causes the spatial inaccessibility of SOC and soil N for soil microbes and enzymes^33,36,37^.

The C storages of the AGB and LB were three times and two times greater, respectively, in the FG than in the GG. Similar results were found that fencing (11 years) significantly increased the C storages of AGB and LB, respectively, comparing with the grazed grassland^12^, and aboveground biomass C storages were about two times greater after 8 years fencing because of fencing increases in soil coverage, plant biodiversity, biological yield, and SWC and nutrients after enclosure construction in slightly degraded steppe grasslands on the Loess Plateau^12,32^. In our study, the C stock of the BGB was not significantly different between the fenced grassland and grazed grassland; the possible reason for this result is that the species diversity of the GG is lower than that in the FG, and fewer roots of species were obtained in comparison to those in the FG, differing from previous studies, in which aboveground biomass C storages were significantly lower in the FG than in the GG^12^. Plant C and N stocks are determined by biomass, and fencing exclusion of livestock resulted in the restoration of the grassland biodiversity, and increased the plant biomass^30,36^. After 30 years of fencing, the nitrogen storages of the aboveground biomass and litter biomass in the FG were greater than those in the GG because of the increased plant biomass and biodiversity. In this study, fencing only affected the vegetation biomass in the 0–10 cm soil layers. In addition, plant roots in the FG were observed in the 60–80 cm soil layer, but there were no roots in the GG within the same soil layer because fencing increased the coverage and species richness of plants, while overgrazing depressed the plant diversity and the growth plant roots, and fencing stimulated plant roots to grow deeper to obtain nutrients and water^38,39^.

In our study, long-term (30 years) grazing exclusion significantly increased SOC in the 10 to 180 cm soil layers and soil N stock in both the GG and FG of the 0–60 cm soil layers, respectively. Previous studies showed that 30 years of fencing dramatically increased the soil C and N stocks in the 0–100 cm soil layer in temperate grassland^33^, and fencing for 11 years notably increased soil C storages in the 0 to 100 cm soil layers and N storages from 0 to 20 cm soil layers in comparison to those in GG^12^, and the C and N stocks of 0–20 soil layers significantly increased with decreasing grazing intensity^39^ because of the increased input of C and N into soils by litter and roots. In the fenced 26 years desert shrubland, the SOC and TN storage in the 0–30 cm soil layer increased by 13.6- and 5.4-fold, respectively^40^. The non-significant difference in SOC stock between the FG and GG in the 0–10 cm soil layer (Fig. 5a) and the non-significant difference in cumulative soil C stock in the 0–30 cm layer of soil between the FG and GG (P >  0.05) (Fig. 6a) were likely due to animal manure input in the GG, leading to more soil C and N accumulation, while livestock trampling also led to greater soil BD in the GG^41^, which led to greater C and N stocks because of increased C and N density. The manure input counteracted the soil C and N accumulation associated with long-term fencing. In the 0–10 cm soil layer, the soil C stock sequestration showed a negative value, indicating that the loss of C from the soil over the past 30 years in the FG (Fig. 7) likely occurred because the plants consumed more soil C than the soil C input by the microbial decomposition of vegetation biomass and litter. Additionally, few studies have focused on deep soil C and N in grasslands. Callesen and James found that deep roots and deep soil layers (0–300 cm) may contribute significantly to nutrient supplies and the soil C storage capacity of temperate and boreal forest ecosystems^42,43^. Li *et al*. estimated that the soil C stock in the 0–300 cm layer could be three times that in the 0–100 cm layer^44^. Wang *et al*. found that more than 50% of soil C storage occurred in the 100–300 cm layer in grasslands and deserts^45^. However, in our study, we estimated that the ecosystem C stocks in the 0–100 cm soil layer in the FG accounted for 41.8% and 58.1% of the ecosystem C stocks in the 0–500 cm and 0–200 cm soil layers, respectively. Meanwhile, ecosystem N stocks in the 0–100 cm soil layer in the FG accounted for 28.0% and 60.6% of the ecosystem N stocks in the 0–500 cm and 0–200 cm soil layers, respectively. Thus, using only the 0–100 cm soil layer to estimate soil C storage would lead to significant underestimation.

The cumulative soil C storages in the 0–80 cm layer, the cumulative soil C storages below 80 cm and the cumulative N storage in 0 to 500 cm soil layer increased dramatically in the fenced grassland compared to that in the grazed grassland, likely resulting from the fencing increasing the vegetation biomass and litter biomass, which supplies a suitable environment for promoting microbial activity and soil texture, and less C input from root-associated sources and possibly greater C output through heterotrophic respiration might have reduced the various SOC stocks^12,30,36^. Fencing promoted high soil coverage and improved the soil moisture, which increased the soil microbial biomass, specifically that of fungi, and restored soils exhibit greater rates of C and N mineralization^46^. Vegetation restoration decreased the C and N losses because of increased soil coverage and plant productivity^47^. However, previous studies showed different results, with no difference in C stocks between FG and GG areas in the 0–10 cm soil layer (P > 0.05), but the C stock was greater for FG in the underlying 10–100 cm soil layers(P < 0.001)compared to that in GG areas^33^. Conversely, grazing exclusion increased the soil C and N storages dramatically in the 0–20 cm soil layers, but not in 20 to 100 cm soil layers^12^. The annual soil C and N sequestration rates indicated that fencing may result in the accumulation of C in the 40 to 180 cm soil layer. The accumulation of soil N in the160–500 cm soil layers likely resulted from soluble N infiltrating deeper into the soil. Overall, fencing significantly improved the C and N stocks in temperate grasslands in northwest China, and fencing was supposed to be a key measure for ecological restoration of degraded grasslands.

## Conclusion

Long-term fencing improved vegetation coverage, height, and ABG and SWC, SOC, and TN content, but it also resulted in decreased pH and BD. Over the 30 years of fencing, the carbon and nitrogen storages of the grassland ecosystem significantly increased to 773.16 and 105.7 Mg ha^−1^, respectively; and in the GG area, the C and N storages of the ecosystem were 453.67 Mg ha^−1^ and 73.83 Mg ha^−1^, respectively. The accumulation of SOC occurred in the 30–180 cm soil layers, reaching 269.6 Mg ha^−1^ after 30 years of fencing, and the accumulation of TN occurred in the 0–60 cm and 160–500 cm soil layers, reaching 5.98 and 22.69 Mg ha^−1^, respectively. Using soil C storages of 0–100 cm soil layer to estimate soil C storage would lead to major underestimates; Although upper soils may be more dynamic in terms of possible C stock changes, the large C stocks in the 100–500 cm soil layers and their dynamics should not be ignored. These findings are important for assessing ecosystem C and N stocks.

## Materials and Methods

### Study area

**Figure 10:**
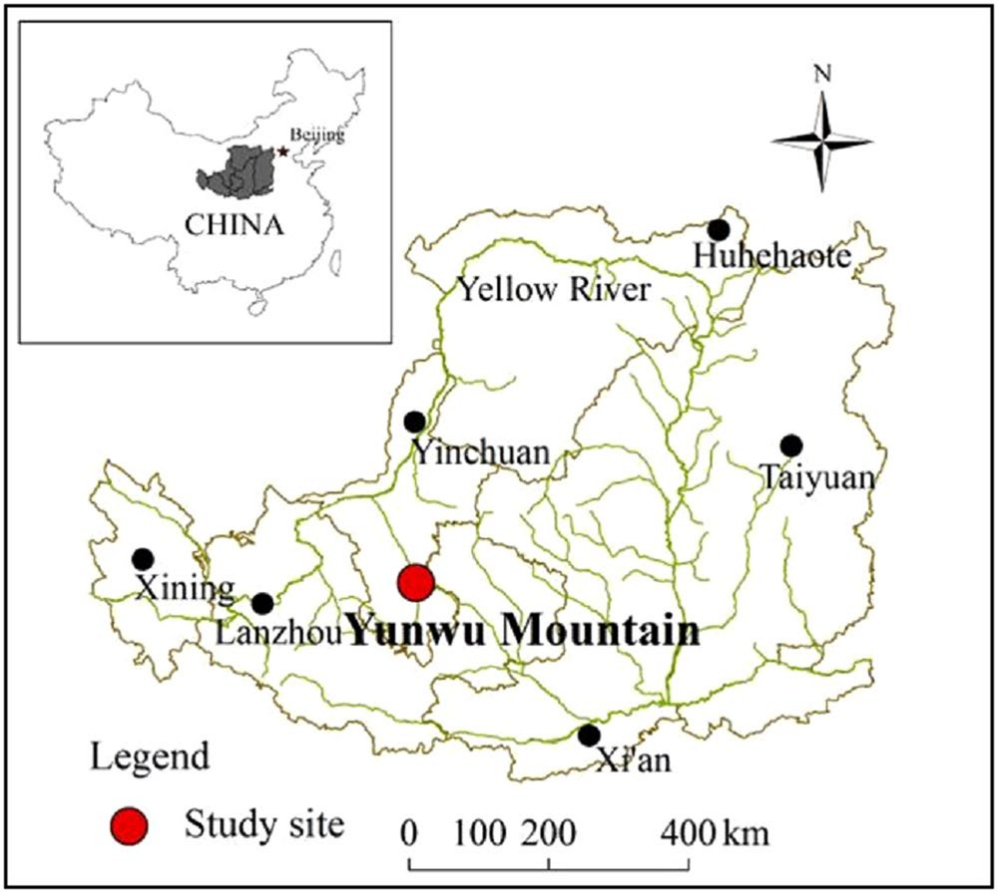
Location of the study site on the Loess Plateau.

The study was conducted in the Yunwu Mountain grassland, Ningxia Province, China (106°16′-106°25′E, 36°09′-36°19′N, 1700–2148 m a.s.l.). Since 1984, the government has implemented exclusionary fencing for ecological restoration, and farmers and livestock are forbidden from disturbing the fenced grassland. The total size of the grassland is approximately 4600 ha, and the areas of the FG and grazed grassland are approximately 3000 ha and 1600 ha, respectively; experimental site information is provided in Table 2 and Fig. 10. The study area, features a hilly landscape, located in semiarid area and the mid-temperate region. The soil was Aeolian and the soil pH value is about 8.3 ± 0.3. The average precipitation from 1960 to 2010 is about 410 mm, of which 75% was received between July and September. The area’s semi-arid temperate continental monsoon climate produces a mean annual temperature of 6.78 °C (1960–2010), a mean annual total of 2518 sunshine hours, a mean annual evaporation of 1600 mm, and 137 frost-free days per year on average, the average depth of the soil is 50 m, and the underground water exists approximately 100 m below the land surface. Meanwhile, the average depth of rainfall infiltration is less than 1 m. At the beginning of fencing, the soil coverage of the grassland was approximately 37%; the dominant species were *Stipabungeana*, *Potentillaacaulis* and *Artemisia frigida*; the average stocking rate was 2.5 sheep ha^−1^; and the grazing season occurred from May to October under nomadic conditions, with the remaining time without grazing occurring from November to March, so the level of grassland degradation was intermediately degraded. After the fencing for grazing exclusion was implemented, the GG remained in the previous grazing management state, but there were no livestock in the FG. There were a few wild animals in the FG, such as rats and rabbits, but the population was small; the livestock in the GG included sheep. After 30 years of fencing, the vegetation in the FG is dominated by *Stipabungeana*, *Stipagrandis*, *Thymus mongolicus*, *Artemisia sacrorum*, and *Potentillaacaulis*, and the soil coverage is approximately 100%. The vegetation in the GG is dominated by *Stipagrandis, Stipabungeana, Artemisia capillaris* and *Artemisia frigida*, and the soil coverage is approximately 36%.

**Table 2:** Experimental site information.

Treatments	Longitude and latitude	Altitude (m)	Gradient(°)	Quadrats	Soil pH
GG	106°24′13.3″E, 36°10′03.6″N	1761	17–19	3	8.4
	106°24′12.8″E, 36°10′00.7″N	1795	0–1	3	8.3
	106°24′11.1″E, 36°09′59.8″N	1788	16–18	3	8.3
FG	106°23′09.9″E, 36°15′03.4″N	2048	13–15	3	8.1
	106°22′53.1″E, 36°15′07.3″N	2077	0–1	3	8.1
	106°23′14.0″E, 36°16′02.9″N	2112	8–10	3	8.0

### Experimental design

The study was performed in August 2014 when the GG and FG grassland biomass were at their peak. A single-factor (two levels, GG and FG) experiment was designed to investigate the differences between the GG and FG. For each level, three 10 m × 10 m plots were set. Within every plot, three 1 m×1 m quadrats were performed only along the diagonal line of the plot (Table 2). The samples from the three quadrats within a single plot composed one sample.

### Biomass measurement

In each FG and GG plot, three quadrats were sampled and composed one sample. All lived aboveground parts of plant were cut and collected, placed them into paper bags, and numbered, as was all litter. To measure the belowground biomass (BGB), a 9-cm-diameter root augur was used to take one soil sample for each depth range of 0–10, 10–20, 20–30, 30–40, 40–60, 60–80 and 80–100 cm in each 1 m×1 m quadrat. Three sub-samples taken from the same layer in the same plot were then mixed to create a single sample, placed into envelopes, and tagged. The root biomass below 100 cm was too small to be measured. The roots found in the soil samples were isolated using a sieve (2 mm, 0.5 mm). All isolated roots were oven dried at 65 °C and weighed.

### Soil sampling and determination

A 51-mm soil sampling drill (S1 Canada) was used to obtain an undisturbed soil core from 0 to 500 cm; soil subsamples from 0 to 40 cm in depth were taken every 10 cm, and from 40 cm to 500 cm, samples were taken every 20 cm. Subsamples from the same layer in the same plot were then mixed together to make one sample, and three mixed samples were created for the GG and FG. The roots and stones were separated from the soil samples by sieving through a 2-mm mesh. The soil samples without roots and debris were air dried and stored for analysing soil physical-chemical indicators.

For the BD first, a 51-mm soil sampling drill (S1 Canada) was used to obtain an undisturbed soil core from 0 to 500 cm, after which the soil BD (g cm^−3^) of the different soil layers (0–10, 10–20, 20–30, 30–40 cm; from 40 to 500 cm, samples were taken every 20 cm) in the undisturbed soil core from the FG and GG plots was measured by soil bulk sampler method, the sampler was 100-cm^3^, the inner diameter of the sampler was 50 mm.

The soil pH value was measured by an acidity agent (PHS-3C pH acidometer, China). The SWC was measured gravimetrically and expressed as the percentage of soil water to dry soil weight. The SOC and plant C were assayed according to the TOC (Vario EL/micro cube, Germany), and TN and plant N content were assayed by the Kjeldahl method^48^.

### Calculation of soil C and N stock

The SOC stock was calculated using the following equation^49^:

$${C}_{S}=\frac{BD\times SOC\times D}{10}$$

Where *C*_*s*_, BD, SOC and D are soil carbon storage (Mg ha^−1^), soil bulk density (g cm^−3^), soil organic carbon (g kg^−1^), and soil depth (cm), respectively. The equation for total N stock, *N*_*s*_, was the same as the *C*_*s*_ equation after substituting the soil TN content for the SOC content.

C sequestration was calculated using the following equation:

$$\begin{array}{rcl}k & = & \Delta {C}_{S}/\Delta Age\\ \Delta {C}_{S} & = & {C}_{FGs}-{C}_{GGs}\end{array}$$

Where *k* is the annual SOC sequestration (Mg ha^−1^yr^−1^), Δ*Age* is fencing years, *C*_*FGs*_ is the SOC stock of the FG (Mg ha^−1^), *C*_*GGs*_ is the stock of the GG(Mg ha^−1^), and Δ*C*_*S*_ is the SOC stock difference between *C*_*FGs*_ and *C*_*GGs*_.

Calculation of vegetation C and N storages using following equations:

$$\begin{array}{rcl}{C}_{s} & = & \frac{\sum {B}_{f}\times {C}_{f}}{100}\\ {N}_{s} & = & \frac{\sum {B}_{f}\times {N}_{f}}{100}\end{array}$$

where *C*_*s*_ is the plant carbon storage, *N*_*s*_ is the plant nitrogen storage (Mg ha^−1^); *B*_*f*_ is the plant biomass (g m^−2^); and *C*_*f*_ and *N*_*f*_ are the carbon and nitrogen content of plant, *f* are ABG, LB and BGB, respectively.

### Ecosystem C and N pools

Ecosystem pools include the C and N of plants and the soil, and the plants include the C and N of the aboveground biomass, litter biomass and belowground biomass.

### Statistical analysis

All data were expressed as the mean ± standard deviation (SD), and a *t*-test was applied to determine the differences in the means between the GG and FG, such as SOC, TN, SWC, BD, plant C and N stocks, and ecosystem C and N sequestration, therefore evaluating the influences of fencing on vegetation, soil, litter and ecosystem characteristics. All statistical analyses were conducted by the software program SAS (SAS Inc. Version 9.2).

## Data Availability

The dataset generated during the current study is available from the corresponding author on reasonable request.
